# *In-vitro* and *In-silico* Haemodynamic Analyses of a Novel Embedded Iliac Branch Device

**DOI:** 10.3389/fcvm.2022.828910

**Published:** 2022-04-05

**Authors:** Shichao Liang, Heyue Jia, Xuehuan Zhang, Wei Guo, Guojing Zhou, Shilong Li, Panpan Yuan, Jiang Xiong, Duanduan Chen

**Affiliations:** ^1^School of Life Science, Beijing Institute of Technology, Beijing, China; ^2^Department of Vascular and Endovascular Surgery, Chinese PLA General Hospital, Beijing, China; ^3^School of Medical Technology, Beijing Institute of Technology, Beijing, China; ^4^Wenzhou Safety (Emergency) Institute of Tianjin University, Tianjin, China

**Keywords:** *in-vitro* experiment, computational fluid dynamics, iliac branch device, embedded branch, mock circulation loop, 3D printing

## Abstract

**Background:**

Iliac branch devices (IBDs) are valid tools for internal iliac artery preservation during endovascular abdominal aortic aneurysm and iliac aneurysm repair. The purpose of this study was to evaluate the effectiveness of a novel IBD with an embedded branch configuration.

**Method:**

A typical iliac artery model was reconstructed, and two models were manufactured using three-dimensional printing technology. The novel IBD was deployed into one iliac artery model by an experienced vascular surgeon. A mock circulation loop (MCL) and a computational fluid dynamics (CFD) simulation were used to investigate the haemodynamic parameters of the iliac models without (Model A) and with (Model B) the IBD. A morphological analysis was conducted using computed tomography angiography and medical endoscopy. The flow distribution rate (FDR) and energy loss (EL) were used to quantify IBD performance.

**Results:**

The FDR of the right internal iliac artery in the MCL of Model A and Model B was 18.88 ± 0.12% and 16.26 ± 0.09%, respectively (*P* = 0.0013). The FDR of the right internal iliac artery in the CFD simulation of Model A and Model B was 17.52 and 14.49%, respectively. The EL of Model A was greater than Model B in both the MCL and the CFD simulation. Compared with Model A, Model B had a larger region (8.46 vs. 3.64%) with a relative residence time of >20 Pa^−1^ at peak systole. Meanwhile, the area where the oscillatory flow index was >0.4 was significantly smaller in Model B than in Model A (0.46 vs. 0.043%). The region with an average wall shear stress of >4 Pa was greater in Model B than in Model A (0 vs. 0.22%).

**Conclusion:**

The MCL and CFD simulation showed that the novel IBD had little impact on the FDR and EL of the iliac artery models. However, the IBD might be an effective tool for the treatment of abdominal aortic/iliac aneurysms that extend into branches. Further investigations are warranted to confirm whether this IBD could be useful in the clinic.

## Introduction

Endovascular aneurysm repair (EVAR) is a feasible and less invasive alternative to open aortic repair for the treatment of abdominal aortic aneurysm (AAA) (1). Although EVAR has a low mortality rate according to randomized clinical trials, long-term failure may occur as the dilation of common iliac arteries (CIAs) which serve as the sealing position at the proximal or distal attachment sites. This complication increases late aneurysm-related complications, such as iliac aneurysm and type Ib endoleak (2). Moreover, an unfavorable iliac anatomy is a major challenge in EVAR. Concomitant CIA aneurysms (CIAAs) are present in 15–40% of patients with AAA, with 70–90% of iliac artery aneurysms affecting the CIA and 10–30% affecting the internal iliac artery (IIAs), which could make EVAR more complicated (3).

Open surgery and traditional endovascular treatments for iliac artery aneurysms often sacrifice the blood supply of the IIA, which may result in buttock or erectile dysfunction, ischemic colitis, and acute limb ischemia (4). Open surgical repair and endovascular bypass are effective methods to transposition or bypass the IIA; however, these procedures are time-consuming. The morbidity and mortality rates of patients who undergo open surgery are also high (5). Therefore, patients with AAA with co-existing iliac artery aneurysms (IIAAs) may require special technical expertise.

Double-barrel endografts (6), external iliac artery (EIA) to IIA stent grafts (7), “sandwich” stent grafts (8), and iliac branch devices (IBDs) (9, 10) have recently emerged as flow-preserving endovascular techniques to address this problem. IBDs are gaining acceptance for the treatment of aorto-iliac aneurysms according to clinical statistics (11). However, most existing IBDs (e.g., Gore IBE, Cook IBD) are designed for use in Western patients based on their anatomy. They may thus not be relevant to patients from the Asia-Pacific region who have smaller iliac arteries (12, 13). Thus, existing IBDs may be restricted in their application in some patients. Austermann et al. (14) described a novel IBD to treat patients with CIAA and IIAA. Our team has also introduced a novel IBD, which was used in Chinese patients in a previous paper (15). However, these IBDs had an overall structure with external vessel branches; thus, their use may be limited in individuals with small vessel dimensions (CIA diameter of no <18 mm) during release.

In this study, we describe a novel IBD with an embedded branch configuration that can be used to treat IIAA. A mock circulation loop (MCL) and a computational fluid dynamics (CFD) simulation, which have previously been used to evaluate multifarious vascular diseases and the usefulness of and interventional device. Both of them had also been used to in the iliac artery disease, and relative devices (16–20). Haemodynamic performance of this novel IBD would be gained by CFD simulation and custom made MCL to evaluate the Flow distribution rate (FDR) and energy loss (EL).

## Materials and Methods

### Models and Stents

**Figure 1:**
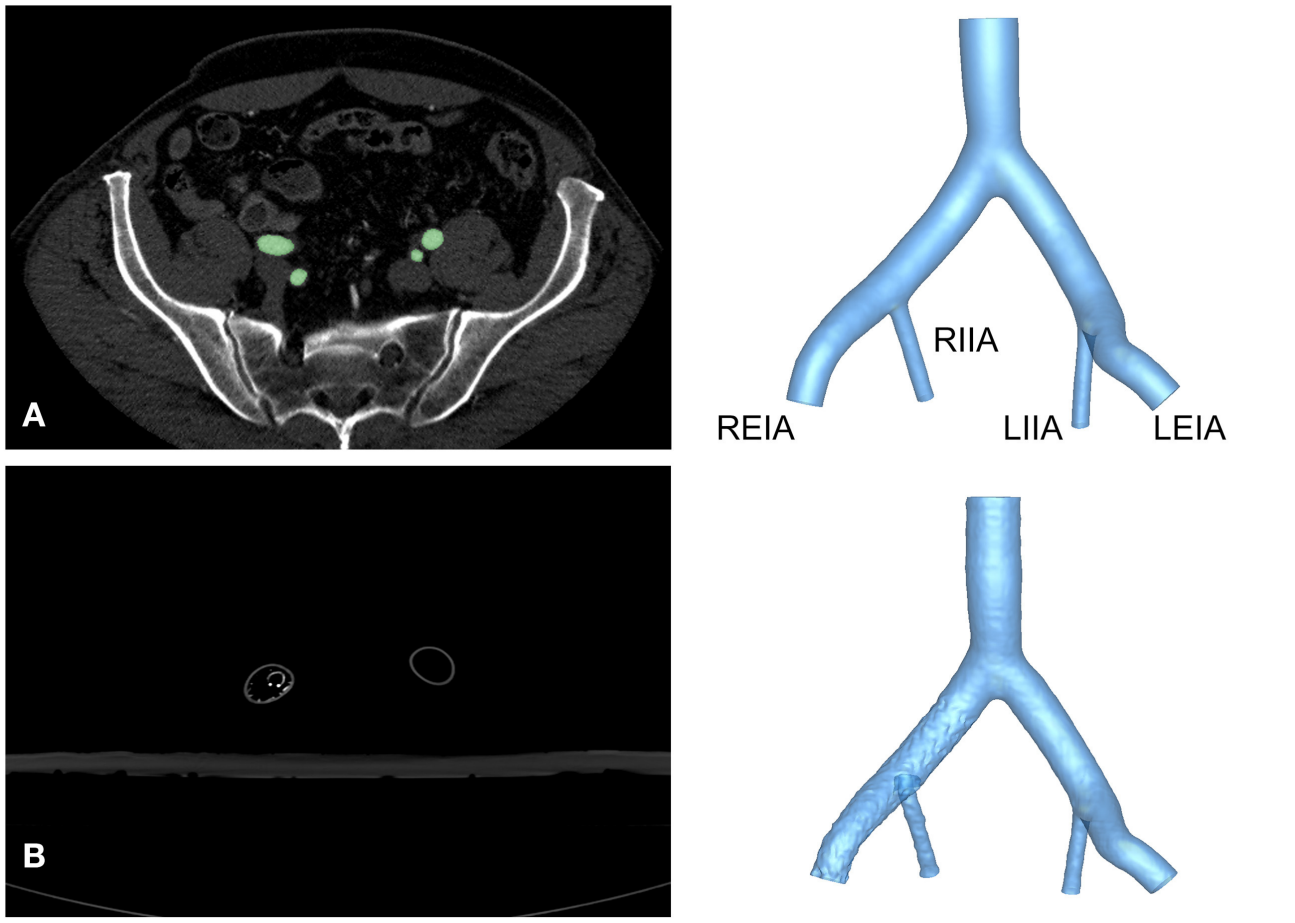
Reconstruction of the iliac artery model. **(A)** Axial view of CTA images from a healthy volunteer and reconstructed structure of Model A. **(B)** Axial view of CTA images and reconstruction of Model B. LEIA, left external iliac artery; LIIA, left internal iliac artery; REIA, right external iliac artery; RIIA, right internal iliac artery.

This study was approved by the Institutional Review Board of the Chinese PLA General Hospital (S201703601). All subjects provided written informed consent. The anatomy of the iliac artery was assessed in subjects with abdominal pain, and arterial disease was ruled out by computed tomography angiography (CTA) using a 16-detector row scanner (Aquilion 16^®^ Toshiba Medical Systems, Japan), as shown in Figure 1. A semi-automatic, threshold-based segmentation tool (Mimics 19.0; Materialize, Belgium) was used to reconstruct the iliac artery model. Then, the iliac artery model was modified by referring to the anatomical characteristics of Chinese patients to obtain a model that was representative of the anatomy of this population. The anatomical size of the iliac artery was shown in Table 1. Three-dimensional (3D) printing software (Magics 19.0; Materialize, Belgium) and computer-aided design software (Solidworks 2018; Dassault Systems, Massachusetts, USA) were used to attain the finial iliac artery model. The anatomical parameters were also shown in Table 1.

**Table 1:** The anatomical parameters of iliac artery model.

**Anatomic parameters**	**Referring dimension**	**Dimension**
The diameter of proximal common iliac artery	25.1 ± 9.4 mm	19.07 mm
The diameter of distal common iliac artery	17.7 ± 7.2 mm	18.03 mm
The diameter of distal external iliac artery	17.7 ± 7.2 mm	14.98 mm
The diameter of internal iliac artery	8.9 ± 6.2 mm	7.43 mm
The length of common iliac artery	57.9 ± 18.1 mm	52.53 mm
The length of external iliac artery	170.8 ± 22.6 mm	71.38 mm
The length of internal iliac artery	44.6 ± 16.5 mm	42.50 mm

**Figure 2:**
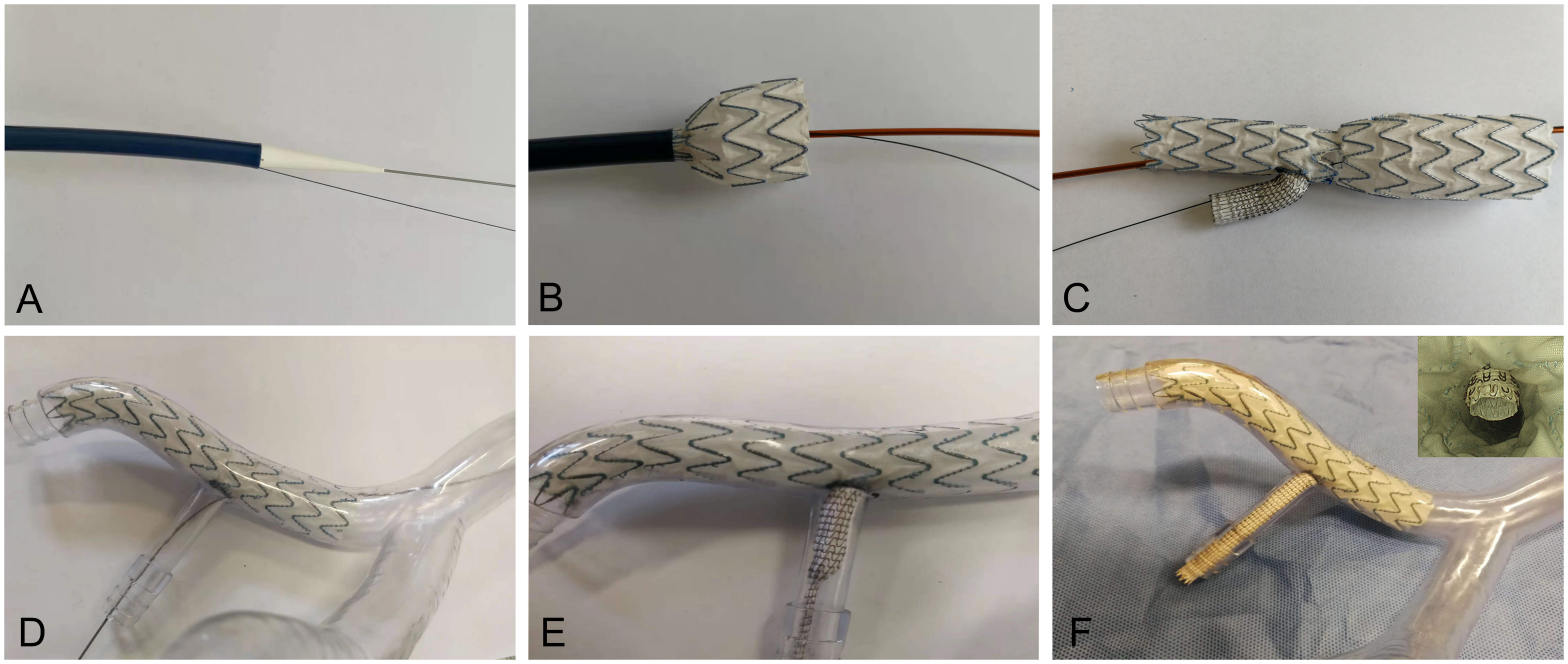
Release of the IBD in the iliac artery model. **(A)** The IBD transporter and guide wire **(B,C)** in the *in-vitro* release simulation. **(D)** The IBD was inserted through the REIA. **(E)** The VIABAHN was implanted into the RIIA. **(F)** The IBD was added into the iliac artery model, and endoscopy was used to visualize the embedded configuration. REIA, right external iliac artery; RIIA, right internal iliac artery; IBD, iliac branch device.

The 3D printing technology was used to manufacture two rigid models with transparent resin material for the *in-vitro* experiment. The iliac artery without the IBD was regarded as Model A. After 3D printing, the IBD configurations were constructed into one iliac artery model (Model B) by an experienced vascular surgeon with the use of regular guide wires and catheters. The novel IBD consisted of two parts: an embedded iliac branch (EIB) and an internal iliac extension (IIE). The EIB was modified by the iliac limb (Medtronic Endurant II; Medtronic, Minneapolis, USA). The proximal and distal diameters were 20 and 16 mm, respectively, and the length was 120 mm. The embedded branch (8 mm in diameter, 10 mm in length) cutting from a covered stent with a diameter of 8 mm (VIABAHN; Gore, Flagstaff, USA). The IIE was a VIABAHN covered stent with a diameter of 8 mm and a length of 10 cm (Figure 2). The EIB was used to open a fenestration with a diameter of 7 mm, and the location of the fenestration was ~5 cm away from the proximal part of the iliac limb. Then, the embedded branch was sutured to the fenestration in an end-to-side anastomosis using continuous hemstick suture (5-0 prolene suture), which is considered to increase the interaction area between the EIB and the IIE to avert sliding, detaching, and other adverse events.

The detailed process of IBD release was shown in Figure 2. The IBD was inserted through the right external iliac artery (REIA) before being partially deployed until the opening window oriented toward the right internal iliac artery (RIIA) (Figure 2D). Then, a guide wire was extended into the RIIA, and the self-expanding stent was implanted through an 8-F catheter to bridge the EIB and the RIIA (Figure 2E). An endoscope was used to investigate coupling between the IIE and the EIB before and after the experiment (Figure 2F).

### Mock Circulation Loop

**Figure 3:**
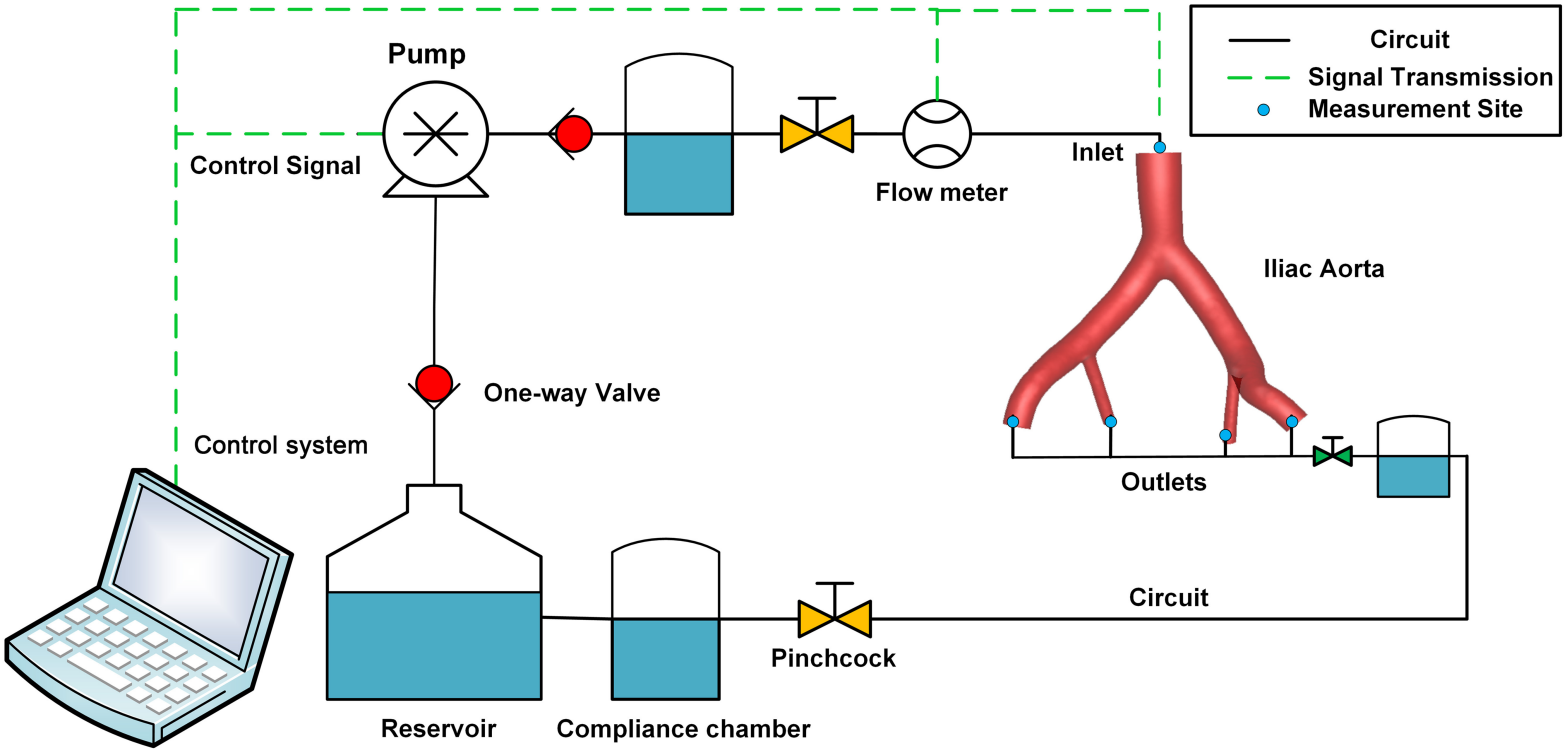
Schematic diagram of the perfusion experiment. The MCL consisted of a pulsatile pump, a control system, a compliance chamber, the iliac artery model, a pinchcock, a one-way valve, a reservoir, pressure sensors, and flow sensors. MCL, mock circulation loop.

The iliac artery model produced by 3D printing was inserted into the custom-made MCL, which had been described in a previous study (21). The MCL consisted of a control system, the Windkessel model, and a reservoir (Figure 3). The blood analog fluid was 40% glycerine in water, with a density of 1,050 kg/m^3^ and a viscosity of 4.1 cP. A hydraulic transducer (HSCDANT005PGAA5; Honeywell, Morristown, NJ, USA) and an ultrasonic flowmeter (ME20PXL291 or ME9PXL1668; Transonic Systems, NY, USA) were used to measure the haemodynamic parameters of the model.

Air was removed from the circulation before the experiment. The MCL reproduced normal resting physiological conditions with a pulsatile flow about 1.8 L per min, and the simulated heart rate was 60 bpm. The FDR of the EIA and the IIA in Model A was set to 60/40. Then, Model B was assembled into the MCL, maintaining the boundary condition settings. After the perfusion experiment, the anatomy of Model B was assessed by CTA (Aquilion 16; Toshiba Medical Systems, Japan), and the reconstructed model was used for the CFD simulation.

### Numerical Simulation and Boundary Conditions

A finite volume solver (CFD-ACE 18.0; ESI Group, Paris, France) was employed to solve the transport equations, including the Navier–Stokes equations, together with the continuity equation of incompressible and Newtonian fluid. The flow in this simulation was assumed to be laminar with the same physical properties as the blood analog fluid in the MCL. The vessel wall was regarded as non-slip and rigid, as is the case in most CFD studies (18, 22). The simulation was carried out for four cardiac cycles using two vessel models with 50 steps per cycle, and the results of the final cycle were selected for post-processing when a converged solution was accomplished. Grid and time step independence tests were also conducted, which proved that the base mesh resolution and time step settings were adequate in this study (23).

The models were then discretized in ICEM-CFD (ANSYS18.0; Canonsburg, USA) with tetrahedral elements in the core region and prismatic cells in the boundary layer near to the aortic wall. The elements varied from 2.5 to 3 million. The flow waveform of the inlet was referenced from the setting data of the MCL. The pulsatile pressure waveforms that were attained by the hydraulic transducers from the MCL were also used as boundary conditions for the outlets.

### Statistical Analysis

The FDR and EL are regarded as key features to evaluate the performance of IBD (24). The FDR of the branches was calculated according to the ratio of branching flow to inflow, while EL was defined as the difference in energy between the inlet and the four outlets in one cardiac cycle (*T* = 1 s). The EL was calculated according to Eq. 1.


(1) $$Energy\;loss\;=\sum_{Inlet}TP*Q-\sum_{outlets}TP{\;}^{*}\;Q$$

where Q indicates the blood flow volume and TP is the total pressure.

Data on the pressure and area flow rate were obtained at the intersected planes, which were 2 mm from the inlet and the four outlets. The detailed positions of the planes were shown in Supplementary Figure 1. Haemodynamic data, including flow pattern, time- averaged wall shear stress (TAWSS), oscillatory flow index (OSI), and relative residence time (RRT), were also analyzed for both models and for the region of interest around the RIIA.

The perfusion experiment was repeated three times, and averaged cycle data were acquired by averaging five cardiac cycles at 500 Hz. The paired *t*-test was used as the statistical method after testing the distribution normality of data obtained by the MCL. Data obtained from the perfusion experiment were presented as mean ± standard deviation, and differences were considered statistically significant when *P* was < 0.05. Differences in Lin's concordance correlation coefficient (LCCC) were used to compare the pressure data obtained from the MCL and the CFD simulation. An LCCC of <0.90 was considered as poor agreement, 0.90–0.95 was considered as reasonable agreement, and >0.95 was considered as good agreement between the two measurements. The Bland–Altman analysis was also used to assess differences between the two methods.

## Results

### Morphological Analysis

The IIE and EIB were tightly fitted after the experiment by endoscopy (Figure 2F). After perfusion, no deformation, collapse, or fitting was noted in the EIB by endoscopy, reflecting the stability of the IBD. Morphological data were obtained from Model B. The length of the extended part of the EIB was 9.12 mm, and the angle between the embedded branch and the EIB was 64.51° (Supplementary Figure 3).

### Comparison of the Haemodynamic Parameters of the MCL and the CFD Simulation

**Figure 4:**
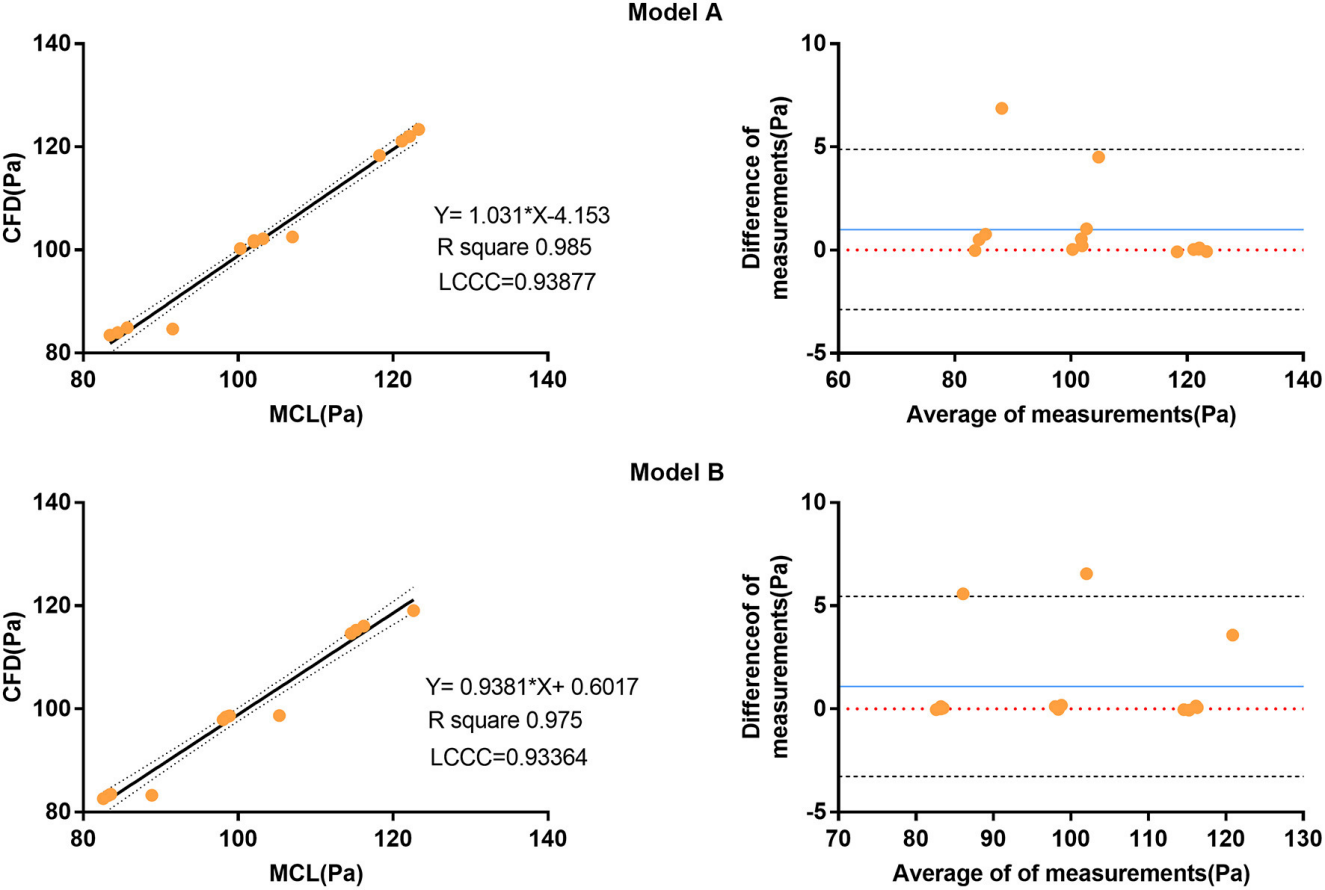
Correlation and Bland-Altman analysis of pressure data from the four outlets (diastolic pressure, time-averaged pressure, and systolic pressure) attained by the MCL and the CFD simulation. The blue solid lines represent the average difference between the pressure data obtained by the MCL and the CFD simulation. The black dotted line represents the 95% CI. CI, confidence interval; MCL, mock circulation loop; CFD, computational fluid dynamics.

**Figure 5:**
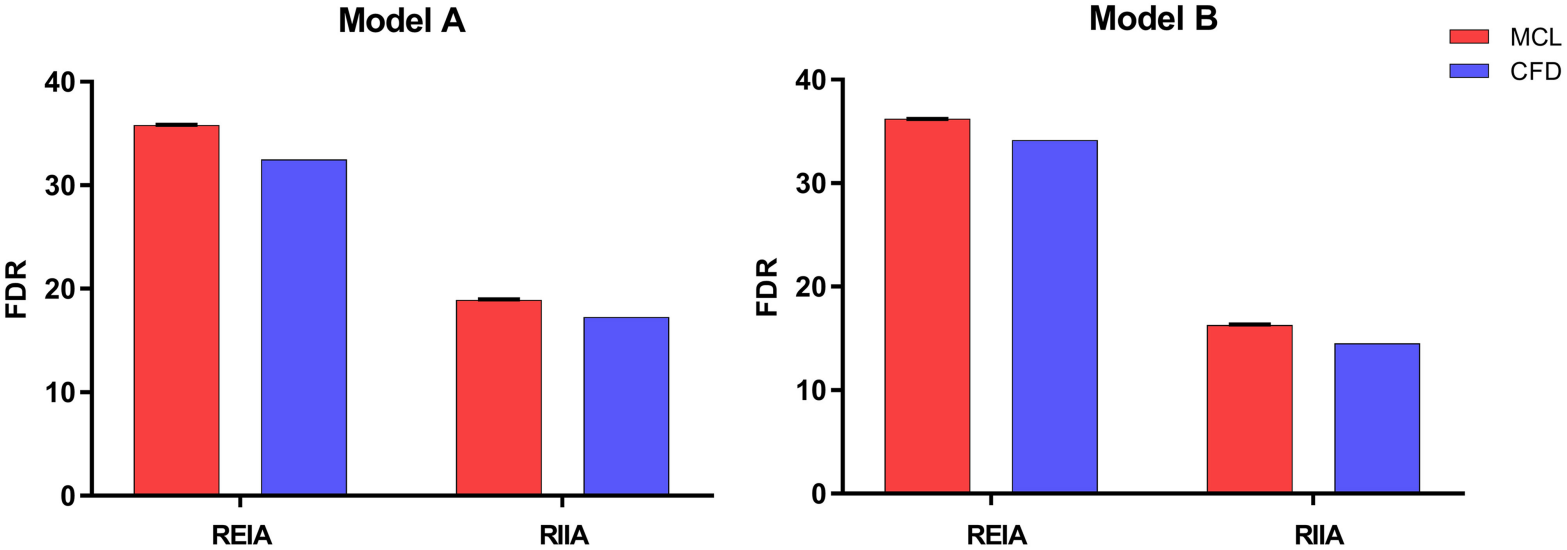
The FDR of the REIA and the RIIA in Model A and Model B attained by two methods under healthy resting conditions. Bar plots indicate the standard deviation. REIA, right external iliac artery; RIIA, right internal iliac artery; FDR, flow distribution rate.

The pressure at the inlet and outlet of Model A and Model B was measured using the MCL and the CFD simulation. The pressure data was presented in Table 2. The time-variant pressure at the inlet was shown in Supplementary Figure 2. Compared with Model A, the pressure in Model B was lower with both the MCL and the CFD simulation. Pressure data from Model A demonstrated an LCCC agreement of 0.9388 [95% confidence interval (CI) 0.9086–0.9592] between the MCL and the CFD simulation, and the mean difference with the Bland–Altman analysis was 0.9975 ± 1.982 Pa. The LCCC agreement of Model B was 0.9336 (95% CI 0.8931–0.9591) between the MCL and the CFD simulation, and the mean difference with the Bland–Altman analysis was 1.093 ± 2.223 Pa (Figure 4). Figure 5 shows the FDR of the REIA and the RIIA in Model A and Model B with the MCL and the CFD simulation. The FDR of the RIIA in Model B was lower than in Model A in the two simulations. Specifically, the FDR of the RIIA in Model A and B with the MCL were 18.88 ± 0.12% and 16.26 ± 0.09%, respectively (*P* = 0.0013), while it was 17.52 and 14.49% with Model A and Model B, respectively, with the CFD simulation. Compared with Model A, the FDR of the REIA increased by 1.21% with the MCL in Model B, while the FDR of the REIA increased by 4.87% with the CFD simulation in Model B.

**Table 2:** The average pressure data at the inlet and four outlets of two models attained by two means.

	**Position**	**Method**	**Systolic pressure (mmHg)**	**Diastolic pressure (mmHg)**	**Time-average pressure (mmHg)**
Model A	Inlet	MCL	123.31	91.54	107.01
		CFD	123.37	84.67	102.51
	REIA	MCL	118.24	83.46	100.29
		CFD	118.37	83.49	100.25
	RIIA	MCL	122.15	84.46	102.06
		CFD	121.75	83.95	101.52
	LEIA	MCL	122.03	85.67	103.20
		CFD	121.99	84.90	102.17
	LIIA	MCL	121.14	84.46	102.04
		CFD	121.11	83.95	101.83
Model B	Inlet	MCL	122.66	88.87	105.31
		CFD	119.08	83.29	98.75
	REIA	MCL	114.57	83.21	98.39
		CFD	114.68	83.20	98.41
	RIIA	MCL	116.36	83.28	98.62
		CFD	115.30	83.16	98.50
	LEIA	MCL	115.23	83.54	98.88
		CFD	115.28	83.50	98.70
	LIIA	MCL	116.23	82.60	98.03
		CFD	116.08	82.63	97.91

*REIA, Right external iliac artery; RIIA, Right internal iliac artery; LEIA, Left external iliac artery; LIIA, Left internal iliac artery*.

The EL with the MCL in Model A and Model B was 3.36 ± 0.19 W and 4.93 ± 0.23 W, respectively (*P* = 0.001). The EL with the CFD simulation in Model A and Model B was 4.237 W and 5.572 W, respectively.

### Haemodynamic Data From the CFD Simulation

**Figure 6:**
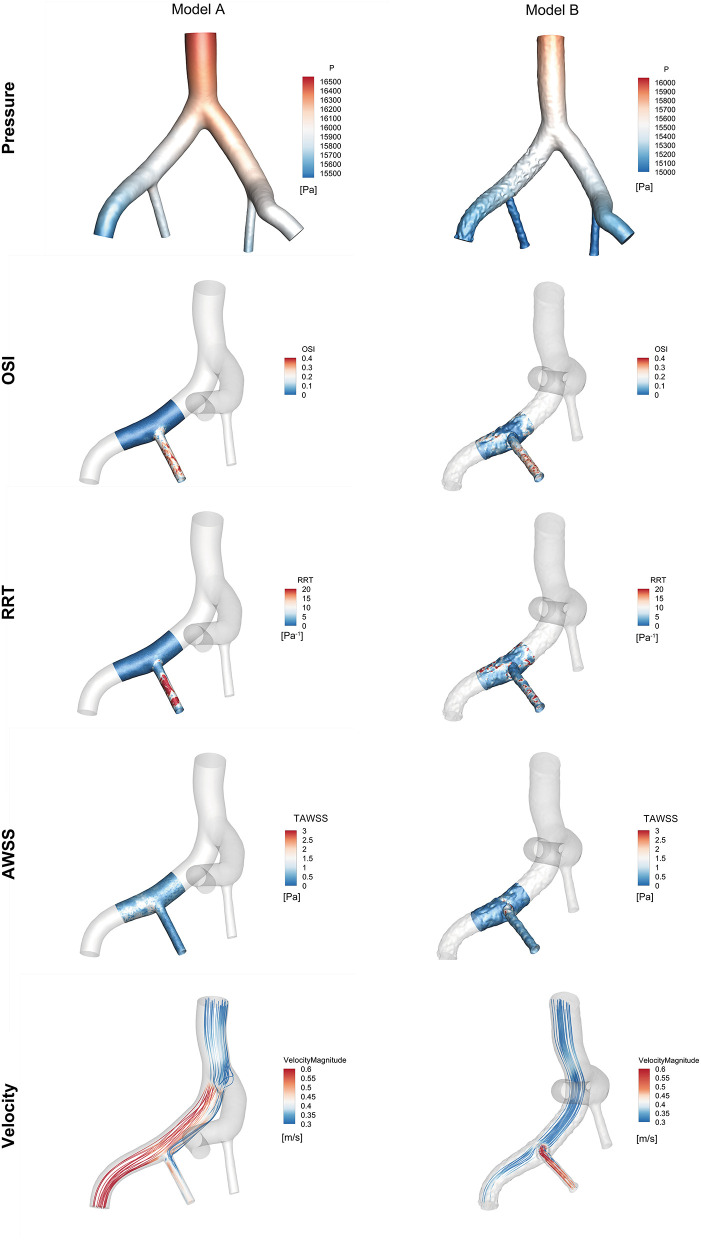
Comparison of haemodynamic data from the region of interest near the right iliac artery in Model A and Model B at peak systole (*t* = 0.16 s).

The haemodynamic results of the CFD simulation at peak systole were shown in Figure 6. Compared with Model A, the region where RRT was >20 Pa^−1^ was smaller in Model B (8.46 vs. 3.64%), as the area where the OSI was >0.4 (0.46% vs. 0.043%). The region with a TAWSS of >4 Pa was greater in Model B than in Model A (0 vs. 0.22%), and the region was concentrated in the protruded part of the stent. The flow streamlines were plotted at peak systole to visualize the flow pattern of the two models. Greater high-velocity flow was observed in the IIE that protruded into the iliac lumen.

## Discussion

Compared with other treatments for IIAA, IBDs have been investigated at specialized centers with simultaneous preservation of ipsilateral revascularization of both the EIA and the IIA. IBDs may provide a new solution for the treatment of AAA and CIAA, and promising mid-term and long-term outcomes with IBDs have been published (25). However, most IBDs were designed based on the morphological parameter of Caucasians. For example, the Branch Endovascular Graft-Iliac Bifurcation device (Zenith; Cook Medical, Bloomington, IN, USA) is indicated in individuals with a CIAA with a diameter of >30 mm or an AAA with a diameter of >55 mm in men or >50 mm in women, with a concomitant CIA aneurysm of >20 mm in diameter (26). Not fulfilling these requirements is the reason for 68.7% of cases of non-eligibility. The diameter of the CIAA in individuals of Asian-Pacific origin was 25–30 mm in the present study. Furthermore, individuals of Asian-Pacific origin have been reported to have a higher risk of complicated CIAA (13). Our team previously reported a polyester IBD with a different proximal diameter, and the mid-term follow-up demonstrated its safety and effectiveness in Chinese individuals. However, the configuration of IBDs may need further improvement. Particularly, the anatomical limitations of IBDs may limit the widespread application of most existing commercial grafts. One reason is that most IBDs always had a uniform configuration which need a rather wide space during the process of releasing in the CIA.

In our study, a novel IBD with a special configuration was introduced. The IBD consisted of an EIB and an IIE. The feature that distinguishes this IBD from other IBDs is the embedded branch design. Some studies had proposed the protrusion configuration in thoracic stent grafts and renal stent grafts to maintain branch perfusion (22, 27). The embedded configuration can reduce the limitations related to the diameter of the CIA by eliminating the integral branch opening.

Endoscopy was used for the morphological analysis of the model. *In-vitro* experiments and *in-silico* simulations had been widely applied to evaluate stent graft performance. They allow the haemodynamic performance of devices to be optimized and effectively reduce the development time. In our research, the effectiveness of this IBD was evaluated using haemodynamic data obtained from the MCL and the CFD simulation.

There was no significant change in pressure drop between Model A and Model B. Specifically, the average pressure drop in the RIIA attained by MCL between two phantoms was 5.79 mmHg, which is a decrease of 4.74%. While that in CFD simulation was 6.45 mmHg, which is a decrease of 5.30%. The average pressure drop in the REIA with the MCL and the CFD was decreased by 3.10 and 3.12%, respectively. At peak systole, the pressure within the embedded stent was lower than in the main stent, indicating that the pressure difference may promote stent contraction or migration.

Adequate perfusion is crucial for maintaining normal organ function. Differences in flow between the two models were assessed based on FDR and EL, which were calculated from the haemodynamic data obtained from the MCL and the CFD simulation. As can be seen from the Figure 5, the FDR of the REIA was increased after implanting the IBD in both simulations. On the contrary, a slight reduction in RIIA blood flow was observed (a decrease of 13.88% with the MCL and a decrease of 17.31% with the CFD simulation). As for the EL the indicator was increased in Model B, which suggests that pulsatile flow was impacted by the protrusion structure. These findings are in concordance with previous research. The novel IBD did not show large changes in FDR and EL, suggesting that it had limited impact on branch perfusion.

A lower OSI was observed at the protrusion region of Model B, suggesting that RIIA flow tended to be stable. The high RRT region of Model B was decreased compared with Model A. The TAWSS in Model B was much greater than in Model A, especially in the protrusion section. It is also interesting to note that the blood around the embedded branch accelerated into the RIIA due to the smaller flow area. Moreover, a relatively low-velocity region was formed at the entrance to the RIIA.

This study has some limitations that should be noted. First, we only evaluated the effectiveness of one novel IBD in this study; thus, we should increase the number of experimental samples in future studies. There are some differences between the physical properties of blood and those of the blood analog used in our experiment. For sure, the models used in the perfusion experiment were rigid vessel models; however, silicone or animal models may have more advantages in terms of their material properties. Subsequently, the CFD simulation was performed on the basis of a rigid wall assumption without vessel elasticity or compliance. This is popular in CFD studies for most clinical purposes. Given these limitations, further investigation is warranted to assess the function of the novel IBD and to determine whether it could be applied in clinical practice.

## Conclusion

In this study, we designed a novel IBD with an embedded configuration to overcome the limitations of the traditional IBD configuration. Haemodynamic performance of this IBD was assessed using a combination of *in-vitro* and *in-silico* methods. In terms of FDR, EL, and pressure drop, the results show that the geometry of the IBD generates mild-to-moderate haemodynamic variations. Ultimately, further investigation is warranted to assess the potential of this IBD for clinical application.

## Data Availability Statement

The original contributions presented in the study are included in the article/Supplementary Material, further inquiries can be directed to the corresponding author/s.

## Ethics Statement

Written informed consent was obtained from the individual(s) for the publication of any potentially identifiable images or data included in this article.

## Author Contributions

DC, SLia, HJ, SLi, and GZ: methodology. XZ, SLia, and HJ: data collection. SLi and PY: analysis. DC and SLia: writing original draft and revision. DC, JX, and WG: supervision. DC and JX: funding acquisition. All authors contributed to the article and approved the submitted version.

## Funding

This work was supported by the National Key R&D Program of China (2018AAA0102600), the Beijing Natural Science Foundation (Z210012, L192010, L192045, and 7212094), the National Natural Science Foundation of China (81970404, 81770465, and 82170498), and the Scientific Research Translational Foundation of Wenzhou Safety (Emergency) Institute of Tianjin University.

## Conflict of Interest

The authors declare that the research was conducted in the absence of any commercial or financial relationships that could be construed as a potential conflict of interest.

## Publisher's Note

All claims expressed in this article are solely those of the authors and do not necessarily represent those of their affiliated organizations, or those of the publisher, the editors and the reviewers. Any product that may be evaluated in this article, or claim that may be made by its manufacturer, is not guaranteed or endorsed by the publisher.

## Abbreviations

IBD: iliac branch device
MCL: mock circulation loop
CFD: computational fluid dynamics
FDR: flow distribution rate
EL: energy loss
EVAR: endovascular aortic repair
AAA: abdominal aortic aneurysm
CIA: common iliac artery
CIAA: concomitant iliac arteries aneurysms
IIA: internal iliac artery
EIA: external iliac artery
EIB: embedded iliac branch
IIE: internal iliac extension
LCCC: Lin's concordance correlation coefficient
TAWSS: time-averaged wall shear stress
OSI: oscillatory shear stress index
RRT: relative residence time.
