# Supplementary material for: In-vitro and In-silico Haemodynamic Analyses of a Novel Embedded Iliac Branch Device
Source: Front Cardiovasc Med. 2022 Apr 5;9:828910. doi: 10.3389/fcvm.2022.828910 (PMC9016111; doi:10.3389/fcvm.2022.828910)
Supplement: Supplementary file 1 [file Data_Sheet_1.docx]

Supplementary Material

**In-Vitro and In-Silico Haemodynamic Analyses of a Novel Embedded Iliac Branch Device**

**Shichao Liang, MS^1^, Heyue Jia, BM^2^, Xuehuan Zhang, MS^1^, Wei Guo, MD, PhD^2^, Guojing Zhou, BS^1^, Shilong Li, BS ^1^, Panpan Yuan, BS ^1^, Jiang Xiong, MD, PhD^2*^, Duanduan Chen, PhD^1,3,4*^**

^1^School of Life Science, Beijing Institute of Technology, China

^2^Department of Vascular and Endovascular Surgery, Chinese PLA General Hospital, China

^3^School of Medical Technology, Beijing Institute of Technology, China

^4^Wenzhou Safety (Emergency) Institute of Tianjin University, China

**S1. Plane locations in Model A and Model B**

To obtain data on the pressure and flow rate at the inlet and outlets in Model A and Model B, relative data were obtained at the intersected planes, which were 2 mm from the inlet and the four outlets. The position was shown in Supplementary Figure 1.


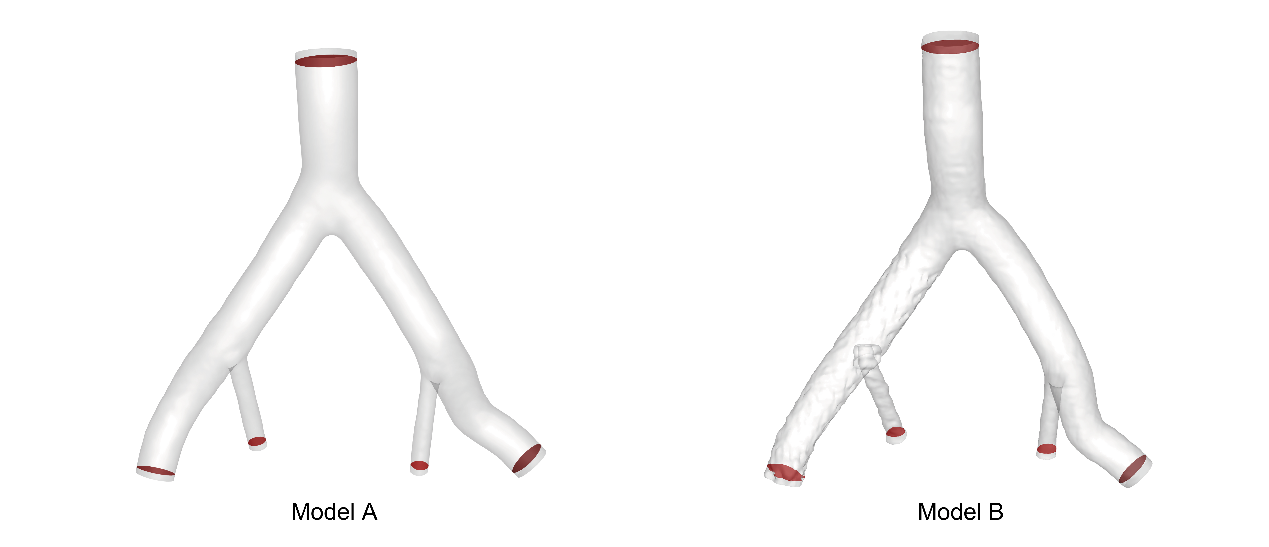


**Supplementary Figure 1.** The intersected planes at the inlet and outlets in Model A and Model B

**S2. Time-variant pressure at the inlet in Model A and Model B**

The time-averaged pressure at the inlet in Model A and Model B were depicted in Supplementary Figure 2.. The pressure attained by mock circulation loop (MCL) demonstrated a lower systolic pressure and a higher diastolic pressure than that from computational fluid dynamics (CFD) simulation. As the MCL could reproduce the compliance of iliac artery, it follows that the pressure waveform obtained with the MCL had a dicrotic notch during diastolic period. Conversely, the vessel wall was regarded as non-slip and rigid in the CFD simulation.


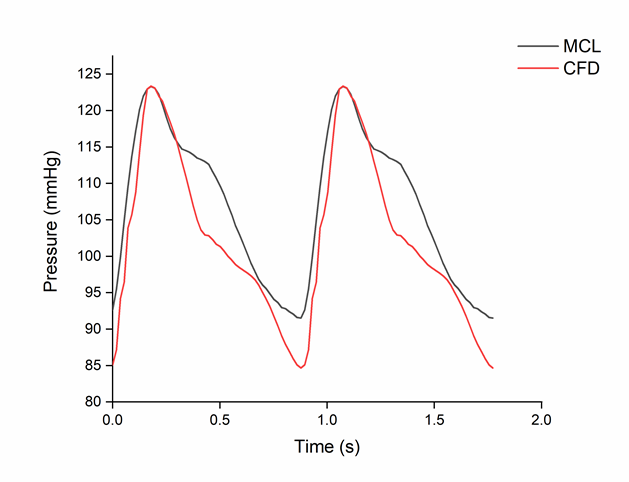


**Supplementary Figure 2.** The pressure waveforms at the inlet in Model A and Model B

**S3. Morphological analysis of Model B**


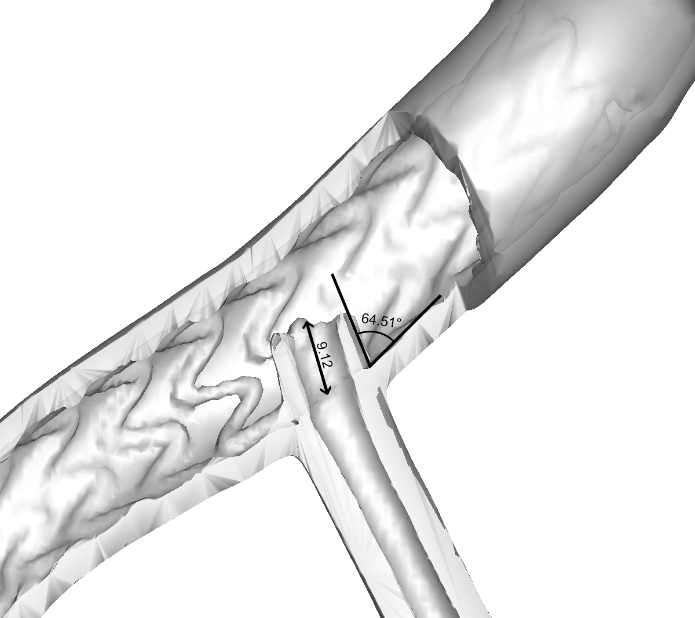
After the perfusion experiment, Model B was reconstructed by computed tomography angiography. The morphological parameters of the embedded part are shown in **Supplementary Figure 3.**

**Supplementary Figure 3.** Morphological analysis of the embedded part
